# Stomach Cancer Prediction Model (SCoPM): An approach to risk stratification in a diverse U.S. population

**DOI:** 10.1371/journal.pone.0303153

**Published:** 2024-05-21

**Authors:** Bechien U. Wu, Elizabeth Y. Dong, Qiaoling Chen, Tiffany Q. Luong, Eva Lustigova, Christie Y. Jeon, Wansu Chen

**Affiliations:** 1 Center for Digestive Health, Department of Gastroenterology, Los Angeles Medical Center, Los Angeles, CA, United States of America; 2 Department of Gastroenterology, Southern California Permanente Medical Group, Los Angeles, CA, United States of America; 3 Department of Research & Evaluation, Southern California Permanente Medical Group, Pasadena, CA, United States of America; 4 Cedars-Sinai Medical Center, Los Angeles, CA, United States of America; Severance Hospital, Yonsei University College of Medicine, REPUBLIC OF KOREA

## Abstract

**Background and aims:**

Population-based screening for gastric cancer (GC) in low prevalence nations is not recommended. The objective of this study was to develop a risk-prediction model to identify high-risk patients who could potentially benefit from targeted screening in a racial/ethnically diverse regional US population.

**Methods:**

We performed a retrospective cohort study from Kaiser Permanente Southern California from January 2008-June 2018 among individuals age ≥50 years. Patients with prior GC or follow-up <30 days were excluded. Censoring occurred at GC, death, age 85 years, disenrollment, end of 5-year follow-up, or study conclusion. Cross-validated LASSO regression models were developed to identify the strongest of 20 candidate predictors (clinical, demographic, and laboratory parameters). Records from 12 of the medical service areas were used for training/initial validation while records from a separate medical service area were used for testing.

**Results:**

1,844,643 individuals formed the study cohort (1,555,392 training and validation, 289,251 testing). Mean age was 61.9 years with 53.3% female. GC incidence was 2.1 (95% CI 2.0–2.2) cases per 10,000 person-years (pyr). Higher incidence was seen with family history: 4.8/10,000 pyr, history of gastric ulcer: 5.3/10,000 pyr, *H*. *pylori*: 3.6/10,000 pyr and anemia: 5.3/10,000 pyr. The final model included age, gender, race/ethnicity, smoking, proton-pump inhibitor, family history of gastric cancer, history of gastric ulcer, *H*. *pylori* infection, and baseline hemoglobin. The means and standard deviations (SD) of c-index in validation and testing datasets were 0.75 (SD 0.03) and 0.76 (SD 0.02), respectively.

**Conclusions:**

This prediction model may serve as an aid for pre-endoscopic assessment of GC risk for identification of a high-risk population that could benefit from targeted screening.

## Introduction

Gastric cancer is the fifth-most common malignancy worldwide, with nearly one million new cases diagnosed each year [1]. In the United States, mortality from gastric cancer remains high, with an estimated 5-year survival rate of only 32.4% [2]. In 2022, the United States is estimated to see over 26,000 cases of gastric cancer and over 11,000 deaths due to gastric cancer [2]. Survival in gastric cancer is highly stage dependent. Recent data from the Surveillance Epidemiology and End Results registry indicate that patients with the earliest stage of gastric cancer (Stage IA) who undergo treatment with surgical resection, are able to achieve 5-year survival rates of up to 94% [3]. Countries with high incidence rates of gastric cancer, such as South Korea and Japan, have implemented nationwide screening programs [4–6]. As a result, data from 2014 showed that 60% of gastric cancer cases detected in Japan were early stage [7]. A meta-analysis in 2016 showed that Asian countries who participated in screening had significantly increased proportions of early-stage gastric cancers [8, 9]. Korea reported promising results from their population-based screening program, with 5-year survival improving to 60% [10, 11].

In contrast, in low prevalence nations such as the United States, where screening is not routinely performed, the diagnosis of gastric cancer is often made after a patient becomes symptomatic, at which point it is more likely to present at an advanced stage. In the United States, up to two-thirds of gastric cancer cases are unresectable at time of diagnosis, with 85% of tumors associated with lymph node metastases [3].

A barrier to population-based screening for gastric cancer in the United States is the overall low incidence of disease. However, significant racial/ethnic disparities exist in the incidence of gastric cancer with Black, Asian, Hispanic, Alaskan native populations being disproportionately impacted [12–14]. Therefore, targeted screening based on established risk factors could be an effective approach for early detection, thereby leading to improved survival. We hypothesized that a risk-prediction model would be able to identify a subgroup of patients at increased risk in whom targeted screening could be beneficial and such application of such a model could lead to more efficient approach to screening compared to traditional risk factor-based strategies.

The aim of the present study was to develop a risk prediction model to identify appropriate high-risk patients who may benefit from screening for gastric cancer. A secondary objective was to compare the potential performance of a model-based approach to risk stratification compared to traditional risk-factor based methods.

## Materials and methods

### Data sources

In this retrospective cohort study, data were obtained from the Research Data Warehouse of the Kaiser Permanente Southern California (KPSC) [15]. KPSC is an integrated healthcare system in which members enroll through the Kaiser Foundation Health Plan for prepaid comprehensive health care. KPSC is one of Kaiser Permanente’s largest regions, providing both inpatient and outpatient services to about 4.8 million members in southern California. The demographic of KPSC is representative of the background population of southern California [16].

KPSC’s Institutional Review Board approved this study and analyses and waived informed consent. Data between the years 2008–2018 were last accessed in April 2022 for research purposes. All authors except CYJ had access to information that could identify individual participants during or after data collection.

### Study participants

**Fig 1:**
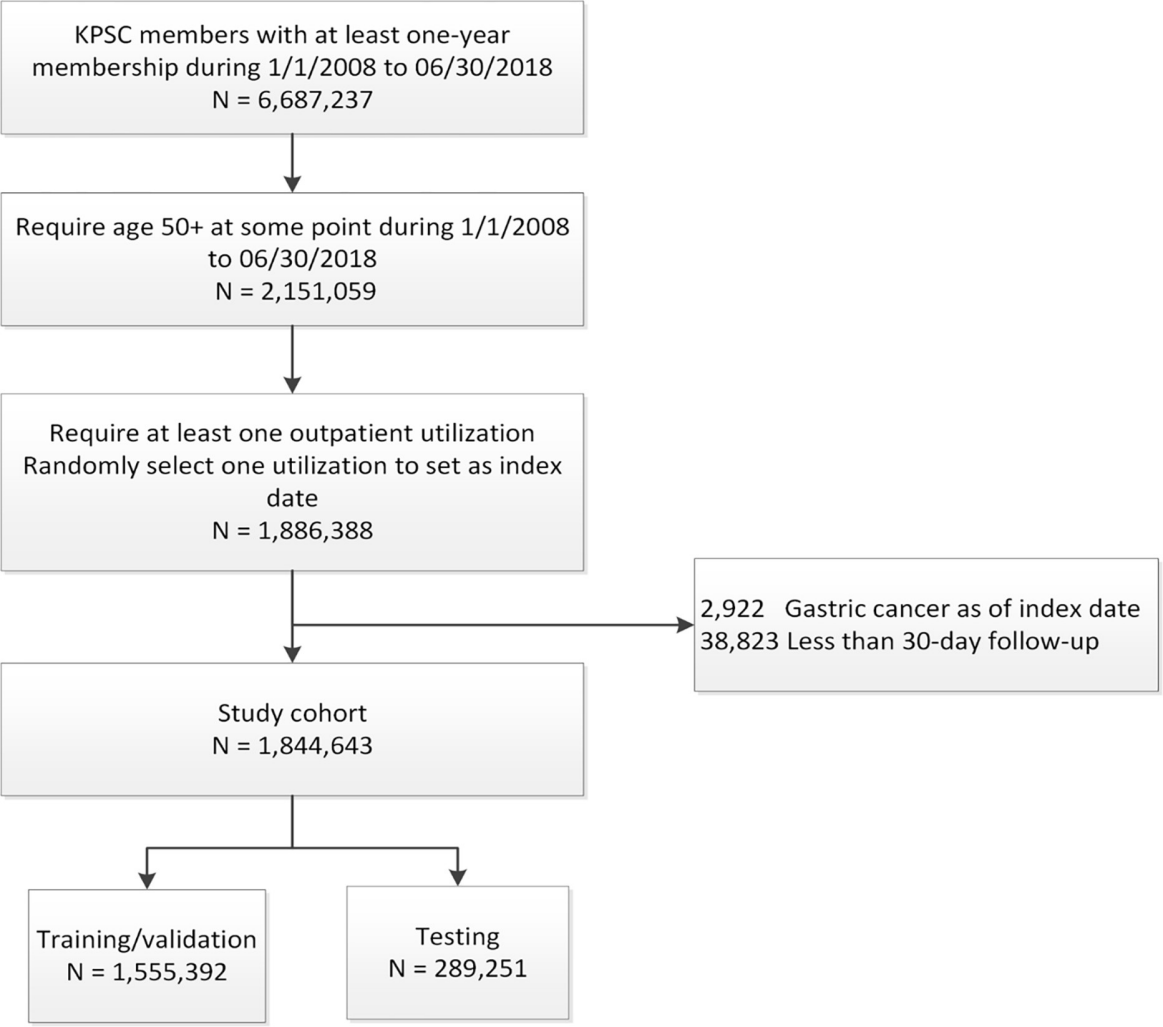
Consort diagram.

We identified a cohort of patients 50–85 years of age who had at least one clinic-based visit at a KPSC medical facility between January 2008 and June 2018. The specific age range was selected based on the proposed target range for potential screening with initiation of screening at age 50 representing a potential cost-effective approach based on prior literature [17]. For patients with multiple qualifying visits during the study visit, one was randomly selected (index date). Patients were excluded if they had a history of gastric cancer prior to the index date or had a follow up period of less than 30 days (Fig 1). Patients were censored at the time of gastric cancer diagnosis, 85 years of age, end of 5-year follow-up period, end of study period on December 31, 2018, disenrollment from health plan, or death.

### Outcome

Incident cases of gastric cancer were identified through an internal prospective cancer registry and the California State Death Master files, captured using International Classification of Diseases, Tenth Revision (ICD-10), C16.0–9.

### Predictors

Covariates that were included in model development included demographic data, lifestyle habits, weight changes, prior history of *Helicobacter pylori* (*H*. *pylori*) infection or gastric ulcer, presence of gastrointestinal symptoms, use of proton pump inhibitor (PPI) therapy, as well as hemoglobin (HGB) and mean corpuscular volume (MCV). Gender was self-reported by patients and represented as a binary outcome, male or female. Race/ethnicity grouping was based on self-reporting by patients at the time of membership enrollment. Racial/ethnic groups were categorized as the following: non-Hispanic White, non-Hispanic Black, Hispanic, Asian/Pacific Islander, and other. Information on family history of gastric cancer was available if previously recorded by a health care provider in the electronic health record. Personal history of *H*. *pylori* infection was ascertained through labs (stool test, antibody test) or diagnosis codes ICD-9 041.86 or ICD-10 B96.81 (problem list). History of gastric ulcer, occurring any time before the index date, was obtained through diagnosis codes.

Smoking status and alcohol intake were obtained through reporting from direct clinical ascertainment in the 12 months prior to the index date. Smoking status was defined as never smoker, former smoker, or current smoker. Alcohol use in the 12 months prior to the index date was reported as yes or no with ‘yes’ indicating regular alcohol consumption. Unknown statuses were also included for both habits.

Use of PPI therapy at any time prior to the index date was assessed through review of the KPSC pharmacy records that include prescription and dispensation information for all health plan members. PPI use was also grouped into no use, new-onset use (within 6 months), or chronic use (beyond 6 months).

We included baseline and prior levels of HGB and MCV, as well as change in both indices as routine laboratory parameters that can serve as early indicators of anemia in the setting of cancer development. Baseline levels were defined by the closest measurement to the index date (including the index date) or within 6 months prior to the index date. Prior levels were defined by the closest measurement to 12 months and occurring within 9–15 months prior to the index date. Change in both indices was calculated as the difference between baseline and prior values.

Baseline body mass index (BMI) was included as a potential predictor with four categories: underweight (less than 18.5 kg/m^2^), normal (18.5 to 25 kg/m^2^), overweight (25 to 30 kg/m^2^), obese (30 or higher kg/m^2^), and unknown. Weight change from 1-year prior (categorized as either gain of over 4.5 kg [10 pounds], gain between 0–4.5 kg [0–10 pounds], loss of ≤ 4.5 kg [0.1–10 pounds], or loss of over 4.5 kg [10 pounds]) was reported but not included as potential predictor, because weight loss is considered a late symptom of cancer. Gastrointestinal symptoms were extracted and reported: abdominal bloating, abdominal pain, fatigue, gastroesophageal reflux, nausea, subjective weight loss, anorexia, early satiety, dyspepsia, dysphagia, melena. However, they were not considered as candidate predictors for the same reason weight loss was not considered i.e., identification of a targeted screening population as opposed to symptomatic evaluation. All reported symptoms were derived from “smart phrases” and/or ICD codes when applicable and were classified as absence of symptoms (asymptomatic), present within 6 months, or present beyond 6 months.

A complete list of variables included in the analyses can be found in S1 Table of S1 File.

### Missing data

‘missRanger’ was applied to impute the missing values if the frequency of missing for a feature was less than 60%. We used predictive mean matching method with k = 3. Laboratory measures or weight-related features with 60% or more missingness or change/change rate measures with 80% or more missingness were not included in the model development process. Ten imputed datasets were generated.

### Statistical analysis

#### Dataset preparation

Records from one KPSC medical service area formed the testing dataset, while those from all other KPSC medical service areas (n = 12) were included for training and validation. Then, we split each of the 10 imputed training/validation datasets into five subsets, each containing 20% of the original imputed dataset (S1 Fig). A total of 50 subsets were prepared for the training and validation process described below.

#### Modeling training and validation

Predictive models were developed using the Cox proportional hazard regression model with least absolute shrinkage and selection operator (LASSO) regularization [18]. Compared to traditional Cox proportional hazard regression models, the models with LASSO regularization reduce the number of predictors and thus prevent overfitting. We developed and validated risk prediction models based on 5-fold cross validation using the 50 training and validation datasets (S2 Table in S1 File) [18]. Of the 50 models derived from the 50 training/internal validation datasets, the one that appeared the most often was selected as the final model.

*Model testing*. Algorithms of the final model was applied to the held-out testing datasets (S2 Table in S1 File). By design, there was no overlap between training, validation, and testing datasets. The discriminative power was evaluated by c-index, averaged across all the relevant testing datasets for cohort members. Calibration was assessed by calibration plots with five risk groups (<50th, 50–74th, 75–90th, 90–94th, 95–100th percentiles). A calibration plot was produced for each winning model.

All analyses were performed using SAS Enterprise Guide 5.1 (SAS Institute, Cary NC) and R. Descriptive statistics were generated for all risk factors for the event of gastric cancer diagnosis. Overall and risk factor-specific incidence rate of gastric cancer and their 95% confidence intervals (CI) were reported.

To assess the potential clinical utility of the final model, we estimated the incidence of gastric cancer among patients based on traditionally defined risk factors (e.g., family history, history of *H*. *pylori*) compared to those based on model-predicted risk. Specifically, we examined the following: incidence among all patients 50–75 years of age, percent true cases in all gastric cancer patients, 5-year incidence rate and 95% CI, time to cancer (in cancer patients only) and number needed to screen to detect one single case.

### Risk estimation

The predicted five-year risk of gastric cancer was estimated based on the individual profile and the baseline hazard. Baseline hazard function was derived from the winning model using Breslow method.

## Results

### Participants

1,844,643 individuals formed the study cohort: 1,555,392 for training and validation with another 289,251 for testing (Fig 1). Mean age of the cohort was 61.9 years (SD 9.37) with 53.3% women (Table 1). The cohort comprised 45.0% non-Hispanic White, 30.0% Hispanic, 10.8% Asian/Pacific Islander and 9.4% non-Hispanic black. 8.4% of the cohort members were current smokers, and 27.3% were former smokers. 33.8% and 34.3% of patients were overweight and obese, respectively. 25.1% of patients used PPI within the 6 months prior to the index date. Symptoms within and beyond the 6 months prior to the index date are found in S3 Table of S1 File. Baseline patient characteristics in the training/validation and testing datasets are reported in S4 Table of S1 File.

**Table 1:** Baseline patient characteristics. N (%) unless otherwise specified. N = 1,844,643.

Patient Characteristics	Value
**Age in years, mean (SD)**	61.9 (9.37)
50–59	920,564 (49.9%)
60–69	543,144 (29.4%)
70+	380,935 (20.7%)
**Sex**	
Female	982,303 (53.3%)
Male	862,340 (46.7%)
**Race/ethnicity**	
Asian/Pacific Islander	198,935 (10.8%)
Black	174,207 (9.4%)
Hispanic	554,220 (30.0%)
White	830,606 (45.0%)
Other/unknown	86,675 (4.7%)
**History of *H*. *pylori* infection**	113,035 (6.1%)
**History of gastric ulcer**	24,932 (22.1%)
**Family history of gastric cancer**	39,081 (2.1%)
**Smoking**	
Non-smoker	1,073,210 (58.2%)
Former smoker	502,828 (27.3%)
Current smoker	154,187 (8.4%)
Unknown	114,418 (6.2%)
**Drinking**	
No	842,637 (45.7%)
Yes	669,616 (36.3%)
Unknown	332,390 (18.0%)
**BMI, mean (SD)**	29.1 (6.15)
Underweight	18,301 (1%)
Normal	413,400 (22.4%)
Overweight	623,996 (33.8%)
Obesity	632,498 (34.3%)
Unknown	156,448 (8.5%)
**Weight change, N = 1,192,754** *	
Median (Q1, Q3)	0 (-4.8, 4.2)
Weight gain > 4.5 kg (10 lbs.)	106,871 (9%)
Weight gain ≤ 4.5 kg or no change	502,773 (42.2%)
Weight loss ≤ 4.5 kg	454,293 (38.1%)
Weight loss > 4.5 kg	128,817 (10.8%)
**Proton pump inhibitors**	
No PPI	1,341,990 (72.8%)
PPI initiated within 6 mos.	462,307 (25.1%)
PPI initiated beyond 6 mos.	40,346 (2.2%)
**Baseline hemoglobin, N = 885,969**	
Median (Q1, Q3)	13.7 (12.7, 14.7)
Normal	652,915 (35.4%)
Abnormal	233,054 (12.6%)
Not tested	958,674 (52%)
**Hemoglobin change, N = 397,476**	
Median (Q1, Q3)	-0.1 (-0.7, 0.5)
**Baseline mean corpuscular volume (MCV), N = 868505**	
Median (Q1, Q3)	90.6 (87.3, 93.7)
Normal	785,776 (42.6%)
Abnormal	82,729 (4.5%)
Not tested	976,138 (52.9%)
**MCV change, N = 382,850**	
Median (Q1, Q3)	0 (-1.4, 1.5)

BMI, body mass index; MCV, mean corpuscular volume; PPI, proton pump inhibitor.

*Weight change is defined as the change in weight within 1 year prior to baseline (baseline– 1 year prior).

A total of 994 patients developed gastric cancer during the study period (S5 Table in S1 File), of which 27.9% were localized, 20.5% regional and 38.4% with involvement of distant organs or distant lymph nodes. The mean age of patients at diagnosis of gastric cancer was 71.1 (SD 9.1) years. Information on cancer site and histology are found in S5 Table of S1 File.

### Incidence of gastric cancer

The overall incidence rate of gastric cancer was 2.1 (95% CI 2.0–2.2) cases per 10,000 person-years (pyr) (Table 2). Higher incidence rates were seen in those with family history of gastric cancer: 4.8 cases (3.6–6.3)/10,000 pyr, history of gastric ulcer: 5.3 (3.9–7.1)/10,000 pyr, *H*. *pylori* infection: 3.6 (3.0–4.4)/10,000 pyr, greater than 10 pound (4.5 kg) weight loss: 3.8 (3.2–4.6)/10,000 pyr, new-onset dyspepsia: 5.6 (4.1–7.7)/10,000 pyr, new-onset dysphagia: 7.3 (5.1–10.3) /10,000 pyr, new-onset PPI use within 6 months: 4.7 (3.5–6.3)/10,000 pyr, and abnormal HGB: 5.3 (4.8–6.0)/10,000 pyr.

**Table 2:** Number of events, total follow-up time, and incidence rate (per 10,000 person-years) and 95% confidence interval.

	Events	Total follow-up in years	Incidence rate (95% CI) (per 10,000 person-years)
**Overall**	994	4,722,862	2.1 (2.0, 2.2)
**Age in years**			
50–59	190	2,263,156	0.8 (0.7, 1.0)
60–69	299	1,459,274	2.0 (1.8, 2.3)
70+	505	1,000,432	5.0 (4.6, 5.5)
**Sex**			
Female	391	2,543,158	1.5 (1.4, 1.7)
Male	603	2,179,704	2.8 (2.6, 3.0)
**Race/ethnicity**			
Asian/Pacific Islander	121	524,271	2.3 (1.9, 2.8)
Black	133	478,201	2.8 (2.3, 3.3)
Hispanic	344	1,357,834	2.5 (2.3, 2.8)
White	383	2,210,387	1.7 (1.6, 1.9)
Other/Unknown	13	152,168	0.9 (0.5, 1.5)
**History of *H*. *pylori* infection**			
No	891	4,438,486	2.0 (1.9, 2.1)
Yes	103	284,376	3.6 (3.0, 4.4)
**History of gastric ulcer**			
No	950	4,639,776	2.0 (1.9, 2.2)
Yes	44	83,086	5.3 (3.9, 7.1)
**Family history of gastric cancer**			
No	943	4,616,461	2.0 (1.9, 2.2)
Yes	51	106,401	4.8 (3.6, 6.3)
**Smoking status**			
Non-smoker	54	312,554	1.7 (1.3, 2.3)
Former smoker	384	1,308,195	2.9 (2.7, 3.2)
Current smoker	452	2,723,947	1.7 (1.5, 1.8)
Unknown	104	378,166	2.8 (2.3, 3.3)
**Weight change** *			
Weight gain > 4.5 kg (10 lbs.)	59	270,552	2.2 (1.7, 2.8)
Weight gain ≤ 4.5 kg or no change	249	1,318,108	1.9 (1.7, 2.1)
Weight loss ≤ 4.5 kg	315	1,182,643	2.7 (2.4, 3.0)
Weight loss > 4.5 kg	120	313,446	3.8 (3.2, 4.6)
Unknown	251	1,638,113	1.5 (1.4, 1.7)
**PPI**			
No PPI	604	3,433,252	1.8 (1.6, 1.9)
PPI initiated within 6 mos.	45	96,131	4.7 (3.5, 6.3)
PPI initiated beyond 6 mos.	345	1,193,479	2.9 (2.6, 3.2)
**Baseline hemoglobin**			
Normal	305	571,984	1.8 (1.6, 2.0)
Abnormal	303	1,712,141	5.3 (4.8, 6.0)
Not tested	386	2,438,737	1.6 (1.4, 1.7)
**Abdominal pain**			
Within 6 mos.	155	430,865	3.6 (3.1, 4.2)
Beyond 6 mos.	310	1,459,580	2.1 (1.9, 2.4)
No symptom	529	2,832,416	1.9 (1.7, 2.0)
**Dyspepsia**			
Within 6 mos.	38	68,218	5.6 (4.1, 7.7)
Beyond 6 mos.	122	489,430	2.5 (2.1, 3.0)
No symptom	834	4,165,214	2.0 (1.9, 2.1)
**Dysphagia**			
Within 6 mos.	31	42,689	7.3 (5.1, 10.3)
Beyond 6 mos.	49	195,053	2.5 (1.9, 3.3)
No symptom	914	4,485,119	2.0 (1.9, 2.2)

PPI, proton pump inhibitor.

*Weight change is defined as the change in weight within 1 year prior to baseline (baseline– 1 year prior).

#### Model development, validation, and testing

**Fig 2:**
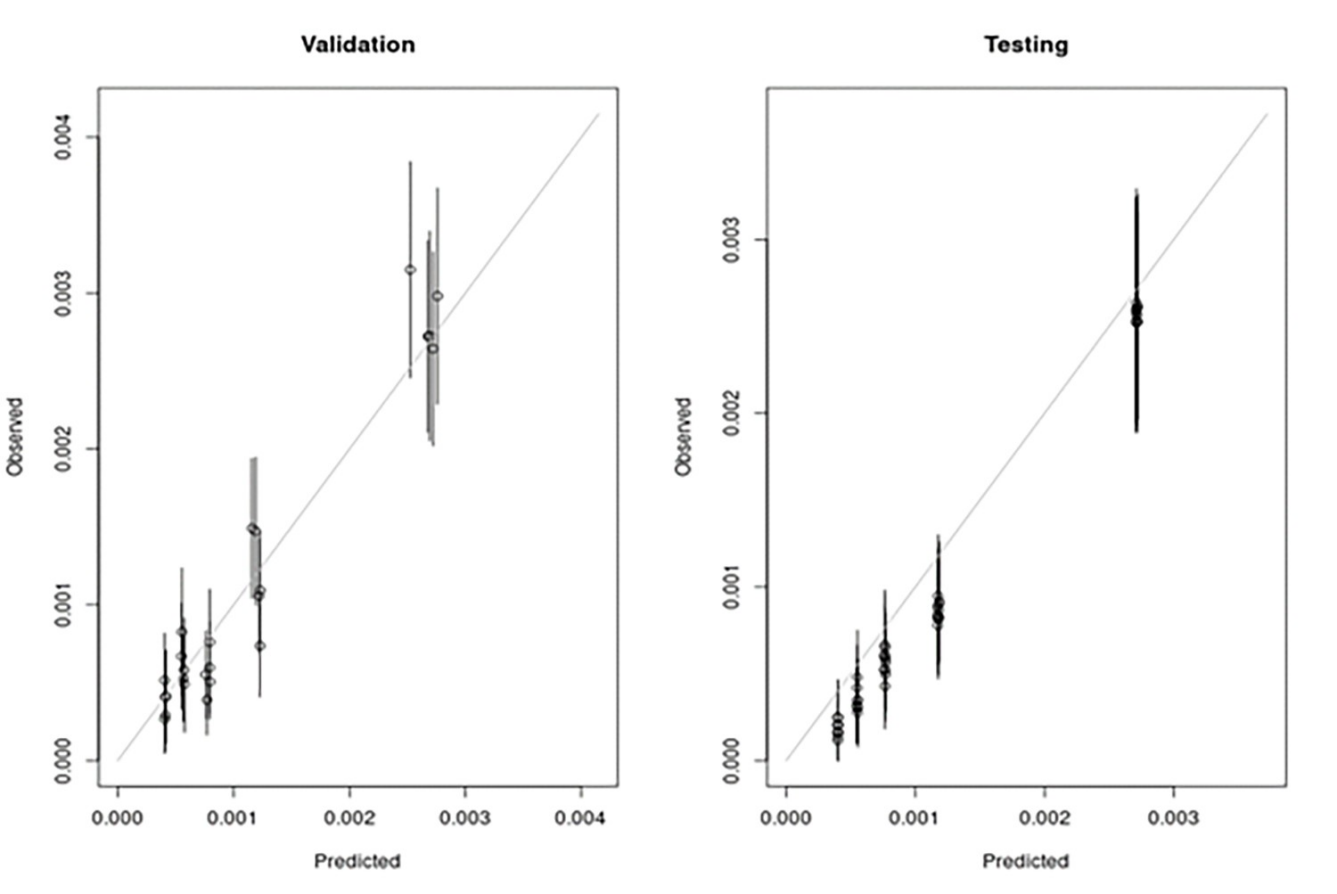
Calibration plot. The means and standard deviations (SD) of c-index based on validation and testing datasets were 0.75 (SD 0.03) and 0.76 (SD 0.02), respectively.

Out of the 50 training datasets, the model including 9 predictors [age, gender, race/ethnicity, smoking, PPI exposure, family history of gastric cancer, history of gastric ulcer, *H*. *pylori* infection, and baseline HGB] was selected as the final model. The means and standard deviations (SD) of c-index based on validation and testing datasets were 0.75 (SD 0.03) and 0.76 (SD 0.02), respectively. The calibration plots for based on both validation and testing datasets are shown in Fig 2.

#### Estimated potential yield of screening for gastric cancer based on traditional risk factors vs. model-predicted risk

Table 3 presents comparison of the potential yield of screening for gastric cancer based on established risk factors as well as according to various thresholds for model-predicted risk. To help frame this comparison, the absolute number and percentage of patients meeting eligibility criteria, proportion (%) of cancers identified according to risk category, 5-year cancer incidence rate as well as number of patients needing to be screened to detect a single cancer (assuming 100% detection rate) are presented. Applying traditional risk factors, 5-year incidence of gastric cancer was highest among patients in the targeted age range with family history (4.0 [95% CI 2.9, 5.5]) cases/10,000 pyr) and prior gastric ulcer (4.6 [3.2, 6.6]/10,000 pyr) with 5.4% and 4.1% of all gastric cancers identified, respectively. Application of model-predicted risk would potentially yield greater efficiency with the highest 2.5 percentile of risk category having 5-year incidence of 8.8 (5.4, 14.3)/10,000 pyr with 13% of cancers represented.

**Table 3:** Targeted screening populations determined by current eligibility criteria and various model-based thresholds.

Traditional risk factors	In all 1,650,416 pts. 50–75 yrs. of age	% in all patients 50–75 years of age	% of all 709 GC cases in the age group	5-yr Incidence rate per 10,000 person-year, 95% CI	Time to GC (year), median (Q1, Q3)	Numbers needed to screen to detect one single case
Age 50–75, Asian	181,541	11.0	11.7	1.7 (1.4, 2.1)	2.0 (0.8, 3.7)	5,812
Age 50–75, Black	154,384	9.4	13.8	2.3 (1.9, 2.8)	1.8 (0.8, 3.0)	4,391
Age 50–75, Hispanic	513,117	31.1	35.4	2.0 (1.8, 2.3)	1.6 (0.6, 2.9)	5,022
Age 50–75, White	718,364	43.5	37.7	1.4 (1.2, 1.5)	1.9 (0.7, 3.2)	7,306
Age 50–75, with *H*. *pylori*	100,449	6.1	10.7	3.0 (2.4, 3.7)	1.3 (0.4, 2.8)	3,350
Age 50–75, with gastric ulcer	24,427	1.5	4.1	4.6 (3.2, 6.6)	1.4 (0.6, 2.6)	2,189
Age 50–75, with family history	34,814	2.1	5.4	4.0 (2.9, 5.5)	1.8 (0.6, 3.3)	2,521
**Model predicted high-risk patients based on validation dataset, age 50–75**	**In all 278,675 pts 50–75 yrs. of age**	**% in all patients 50–75 years of age**	**% of all 123 GC cases in the age group**	**5-yr Incidence rate per 10,000 person-year, 95% CI**	**Time to GC (year), median (Q1, Q3)**	**Numbers needed to screen to detect one case**
Predicted risk ≥0.0012432874^a^	55,735	20	49.6	3.9 (3.1, 5.1)	1.9 (0.5, 3.1)	2,544
Predicted risk ≥0.0014283849^b^	41,800	15	40.7	4.3 (3.3, 5.7)	1.6 (0.5, 2.8)	2,329
Predicted risk ≥0.001680073^c^	27,868	10	32.5	5.2 (3.8, 7.1)	1.4 (0.5, 2.7)	1,932
Predicted risk ≥0.0021037118^d^	13,933	5	17.9	5.8 (3.8, 8.8)	1.5 (0.5, 2.4)	1,721
Predicted risk ≥0.0025205328^e^	6,967	2.5	13.0	8.8 (5.4, 14.3)	1.5 (0.4, 2.3)	1,141

a/b/c/d/e: Risk threshold that identifies top 20/15/10/5/2.5 percentile of patients. GC: Gastric cancer.

## Discussion

Based on data from a racially/ethnically diverse, regional integrated health care system in the United States, we developed a risk prediction model for gastric cancer. The model included a total of 9 routinely available parameters: age, sex, racial/ethnic background, family history, smoking status, *H*. *pylori* infection, PPI usage, as well as HGB value. These factors can be easily obtained from patient interview or abstracted from the electronic medical record. The prediction tool demonstrated a potentially more efficient approach to targeted screening for gastric cancer compared to reliance on traditional risk factors.

Within the United States, gastric cancer currently ranks 15^th^ of the most common malignancies [2]. The estimated number of annual U.S. deaths from gastric cancer is 11,000, with a 5-year survival rate of 32% qualifying it as a recalcitrant cancer [2]. A significant factor in the overall poor survival in the US is the advanced stage at diagnosis given the absence of population-based screening [19]. In contrast, developed countries with high prevalence rates of gastric cancer such as South Korea or Japan that have implemented widespread endoscopic screening there has been a corresponding reduction in gastric cancer-related mortality [4–6].

A targeted approach to screening of high-risk populations is likely to provide an effective strategy for early detection of gastric cancer in low prevalence populations such as the US. A previous study using a Markov model simulating gastric cancer screening analyzing quality-adjusted life years found that screening with upper endoscopy and biopsy was potentially cost effective in Asian, Hispanic, and non-Hispanic Black populations in the US [17], reinforcing previous data on racial/ethnic disparities in the incidence of gastric cancer [12]. The authors proposed an initial screening upper endoscopy with screening colonoscopy at age 50 years, with repeat exams only if precursor lesions such as intestinal metaplasia or dysplasia were discovered from biopsies obtained during index exam.

Previous efforts in developing prediction models for gastric cancer have originated primarily from high prevalence countries [20]. Among lower prevalence nations, the European Prospective Investigation into Cancer and Nutrition cohort study involved multiple European countries, establishing a composite “healthy-lifestyle index” which was made up of modifiable lifestyle factors such as smoking status, weight, diet quality, and alcohol consumption [21]. In addition, a recently developed model based on a case-control study in the Veterans Affairs health system showed promise for identification of gastric intestinal metaplasia [22]. The present study expands on these prior efforts by focusing on risk of gastric cancer in a racially/ethnically diverse low-prevalence regional population in the United States using exclusively parameters routinely available in clinical practice. By comparing the model performance to patient selection based on traditional risk factors, we further demonstrated the potential for increased efficiency using the model-based approach with the ability to detect a greater proportion of cancers (sensitivity) as well as further enrichment (increased positive-predictive value).

In the present study we relied on established risk factors for model development to ensure face validity. Demographic factors such as older age, male sex and non-White racial/ethnic groups experience higher rates of stomach cancer in the US [2]. Likewise, *H*. *pylori* infection [23], family history of gastric cancer in a first-degree relative [24], smoking [25], long-term proton pump inhibitor use [26] as well as prior history of gastric ulcer [27] are each well-established risk factors for stomach cancer. In contrast to these risk factors, the presence of a low hemoglobin may actually represent an early sign of gastric cancer with iron-deficiency anemia that may occur in the setting of atrophic gastritis or potentially directly from occult gastrointestinal hemorrhage from a gastric neoplasia.

The present study has several limitations. The low incidence of cases of gastric cancer during the study period limited the ability to develop more robust, empirically derived data-driven models. We therefore incorporated established risk factors into the current prediction model to limit potential overfitting. Also, given the retrospective nature of the study, we were unable to evaluate the role of diet in determining risk of gastric cancer as certain types of diet including preserved foods or nitrates is an established risk factor for development of gastric cancer [28]. We were also unable to evaluate the role of physical activity given lack of reliable data in the electronic health record during the study years. We did not include histologic data such as presence of atrophic gastritis or intestinal metaplasia as part of our model, as our model is intended to be applied as a pre-endoscopic tool in decision-making regarding initiation of screening. Finally, we did not evaluate the performance of the model within each racial and ethnic group in the current study. Future studies may expand the work and develop race- or ethnicity-specific models if needed.

In conclusion, we have developed a prediction model for gastric cancer based on data from the United States within a diverse racial/ethnic and socioeconomic population. Application of the model may be used to identify patients who are at higher risk for gastric cancer within a 5-year time frame representing a reasonable time frame to undergo endoscopic testing and surveillance. Such an approach to patient selection may be more widely applicable with the widespread adoption of electronic health systems providing an opportunity for targeted screening and prevention for gastric cancer in low prevalence regions such as the United States.

## Supporting information

S1 Checklist STROBE statement—Checklist of items that should be included in reports of observational studies. (DOCX)

S1 Fig Preparation of training and validation datasets. (DOCX)

S1 File Supporting information. (DOCX)

## Abbreviations

BMI: body mass index
CI: confidence interval
GC: gastric cancer
HGB: hemoglobin
ICD-9-CM: Ninth Revision of International Classification of Diseases, Clinical Modification
ICD-10-CM: Tenth Revision of International Classification of Diseases, Clinical Modification
KPSC: Kaiser Permanente Southern California
LASSO: least absolute shrinkage and selection operator
PPI: proton pump inhibitors
PYR: person-year
SD: standard deviation
SE: standard error
