# Supplementary material for: Stomach Cancer Prediction Model (SCoPM): An approach to risk stratification in a diverse U.S. population
Source: PLoS One. 2024 May 21;19(5):e0303153. doi: 10.1371/journal.pone.0303153 (PMC11108155; doi:10.1371/journal.pone.0303153)
Supplement: S1 Checklist — (DOCX) [file pone.0303153.s001.docx]

STROBE Statement—checklist of items that should be included in reports of observational studies

|  | Item No. | Recommendation | Page  No. | Relevant text from manuscript |
| --- | --- | --- | --- | --- |
| **Title and abstract** | 1 | (*a*) Indicate the study’s design with a commonly used term in the title or the abstract | 4 | “We performed a retrospective cohort study from Kaiser Permanente Southern California from January 2008-June 2018 among individuals age ≥50 years.” |
|  |  | (*b*) Provide in the abstract an informative and balanced summary of what was done and what was found | 4-5 | “Abstract  Background and aims  …  Conclusions  This prediction model may serve as an aid for pre-endoscopic assessment of GC risk for identification of a high-risk population that could benefit from targeted screening.” |
| Introduction | | | |  |
| Background/rationale | 2 | Explain the scientific background and rationale for the investigation being reported | 6-7 | Introduction  Gastric cancer is the fifth-most common malignancy worldwide, with nearly one million new cases diagnosed each year.(1) … However, significant racial/ethnic disparities exist in the incidence of gastric cancer with Black, Asian, Hispanic, Alaskan native populations being disproportionately impacted.(12-14) Therefore, targeted screening based on established risk factors could be an effective approach for early detection, thereby leading to improved survival.” |
| Objectives | 3 | State specific objectives, including any prespecified hypotheses | 7 | “We hypothesized that a risk-prediction model would be able to identify a subgroup of patients at increased risk in whom targeted screening could be beneficial and such application of such a model could lead to more efficient approach to screening compared to traditional risk factor-based strategies.  The aim of the present study was to develop a risk prediction model to identify appropriate high-risk patients who may benefit from screening for gastric cancer. A secondary objective was to compare the potential performance of a model-based approach to risk stratification compared to traditional risk-factor based methods.” |
| Methods | | | |  |
| Study design | 4 | Present key elements of study design early in the paper | 8 | “In this retrospective cohort study, data were obtained from the Research Data Warehouse of from the Kaiser Permanente Southern California (KPSC).” |
| Setting | 5 | Describe the setting, locations, and relevant dates, including periods of recruitment, exposure, follow-up, and data collection | 8 | “In this retrospective cohort study, data were obtained from the Research Data Warehouse of from the Kaiser Permanente Southern California (KPSC). KPSC is an integrated healthcare system in which members enroll through the Kaiser Foundation Health Plan for prepaid comprehensive health care. KPSC is one of Kaiser Permanente’s largest regions, providing both inpatient and outpatient services to about 4.8 million members in southern California. The demographic of KPSC is representative of the background population of southern California.(15)”  “We identified a cohort of patients 50-85 years of age who had at least one clinic-based visit at a KPSC medical facility between January 2008 and June 2018.”  “Patients were censored at the time of gastric cancer diagnosis, 85 years of age, end of 5-year follow-up period, end of study period, disenrollment from health plan, or death.” |
| Participants | 6 | (*a*) *Cohort study*—Give the eligibility criteria, and the sources and methods of selection of participants. Describe methods of follow-up  *Case-control study*—Give the eligibility criteria, and the sources and methods of case ascertainment and control selection. Give the rationale for the choice of cases and controls  *Cross-sectional study*—Give the eligibility criteria, and the sources and methods of selection of participants | 8 | “We identified a cohort of patients 50-85 years of age who had at least one clinic-based visit at a KPSC medical facility between January 2008 and June 2018.”  “Patients were censored at the time of gastric cancer diagnosis, 85 years of age, end of 5-year follow-up period, end of study period, disenrollment from health plan, or death.” |
|  |  | (*b*) *Cohort study*—For matched studies, give matching criteria and number of exposed and unexposed  *Case-control study*—For matched studies, give matching criteria and the number of controls per case | N/A | N/A |
| Variables | 7 | Clearly define all outcomes, exposures, predictors, potential confounders, and effect modifiers. Give diagnostic criteria, if applicable | 8-9 | “Outcome  Incident cases of gastric cancer were identified through an internal prospective cancer registry and the California State Death Master files, captured using International Classification of Diseases, Tenth Revision (ICD-10), C16.0-9.  Predictors  Covariates that were included in model development included demographic data, lifestyle habits, weight changes, prior history of Helicobacter pylori (H. pylori) infection or gastric ulcer, presence of gastrointestinal symptoms, use of proton pump inhibitor (PPI) therapy, as well as routine lab parameters. Gender was self-reported by patients and represented as a binary outcome, male or female. Race/ethnicity grouping was based on self-reporting by patients at the time of membership enrollment. Racial/ethnic groups were categorized as the following: non-Hispanic white, non-Hispanic black, Hispanic, Asian/Pacific Islander, and other. Information on family history of gastric cancer was available if previously recorded by a health care provider in the electronic health record. Personal history of H. pylori infection was ascertained through diagnosis codes ICD-9 041.86 or ICD-10 B96.81. History of gastric ulcer was obtained through diagnosis codes.  Smoking status and alcohol intake were obtained through reporting from direct clinical ascertainment. Smoking status was defined as never smoker, former smoker, or current smoker. Alcohol use was reported as either active use, or no active use. Unknown statuses were also included for both habits.  Use of PPI therapy was assessed through review of the pharmacy records. PPI use was also grouped into no use, new-onset use (within 6 months), or chronic use (beyond 6 months).  Laboratory biomarkers included were hemoglobin (HGB) level, mean corpuscular volume, as well as change in both indices.  Body mass index (BMI) was included as a potential predictor with four categories: underweight (less than 18.5 kg/m2), normal (18.5 to 25 kg/m2), overweight (25 to 30 kg/m2), obese (30 or higher kg/m2), and unknown. Weight change from 1-year prior (categorized as either gain of over 10 pounds, gain between 0-10 pounds, loss of 0.1-10 pounds, or loss of over 10 pounds) was reported but not included as potential predictor, because weight loss is considered a late symptom of cancer. Gastrointestinal symptoms were extracted and reported: abdominal bloating, abdominal pain, fatigue, gastroesophageal reflux, nausea, subjective weight loss, anorexia, early satiety, dyspepsia, dysphagia, melena. However, they were not considered as candidate predictors for the same reason weight loss was not considered i.e., identification of a targeted screening population as opposed to symptomatic evaluation. All reported symptoms were derived from “smart phrases” and ICD codes when applicable, and were classified as absence of symptoms (asymptomatic), present within 6 months, or present beyond 6 months.  A complete list of variables included in the analyses can be found in Table S1.” |
| Data sources/ measurement | 8* | For each variable of interest, give sources of data and details of methods of assessment (measurement). Describe comparability of assessment methods if there is more than one group | 8-9 | “Outcome  Incident cases of gastric cancer were identified through an internal prospective cancer registry and the California State Death Master files, captured using International Classification of Diseases, Tenth Revision (ICD-10), C16.0-9.  Predictors  Covariates that were included in model development included demographic data, lifestyle habits, weight changes, prior history of Helicobacter pylori (H. pylori) infection or gastric ulcer, presence of gastrointestinal symptoms, use of proton pump inhibitor (PPI) therapy, as well as routine lab parameters. Gender was self-reported by patients and represented as a binary outcome, male or female. Race/ethnicity grouping was based on self-reporting by patients at the time of membership enrollment. Racial/ethnic groups were categorized as the following: non-Hispanic white, non-Hispanic black, Hispanic, Asian/Pacific Islander, and other. Information on family history of gastric cancer was available if previously recorded by a health care provider in the electronic health record. Personal history of H. pylori infection was ascertained through diagnosis codes ICD-9 041.86 or ICD-10 B96.81. History of gastric ulcer was obtained through diagnosis codes.  Smoking status and alcohol intake were obtained through reporting from direct clinical ascertainment. Smoking status was defined as never smoker, former smoker, or current smoker. Alcohol use was reported as either active use, or no active use. Unknown statuses were also included for both habits.  Use of PPI therapy was assessed through review of the pharmacy records. PPI use was also grouped into no use, new-onset use (within 6 months), or chronic use (beyond 6 months).  Laboratory biomarkers included were hemoglobin (HGB) level, mean corpuscular volume, as well as change in both indices.  Body mass index (BMI) was included as a potential predictor with four categories: underweight (less than 18.5 kg/m2), normal (18.5 to 25 kg/m2), overweight (25 to 30 kg/m2), obese (30 or higher kg/m2), and unknown. Weight change from 1-year prior (categorized as either gain of over 10 pounds, gain between 0-10 pounds, loss of 0.1-10 pounds, or loss of over 10 pounds) was reported but not included as potential predictor, because weight loss is considered a late symptom of cancer. Gastrointestinal symptoms were extracted and reported: abdominal bloating, abdominal pain, fatigue, gastroesophageal reflux, nausea, subjective weight loss, anorexia, early satiety, dyspepsia, dysphagia, melena. However, they were not considered as candidate predictors for the same reason weight loss was not considered i.e., identification of a targeted screening population as opposed to symptomatic evaluation. All reported symptoms were derived from “smart phrases” and ICD codes when applicable, and were classified as absence of symptoms (asymptomatic), present within 6 months, or present beyond 6 months.  A complete list of variables included in the analyses can be found in Table S1.” |
| Bias | 9 | Describe any efforts to address potential sources of bias | 20 | “The present study has several limitations. Given the retrospective nature of the study, we were unable to evaluate the role of diet in determining risk of gastric cancer as certain types of diet including preserved foods or nitrates is an established risk factor for development of gastric cancer.(21) We did not include histologic data such as presence of atrophic gastritis or intestinal metaplasia as part of our model, as our model is intended to be applied as a pre-endoscopic tool in decision-making regarding initiation of screening.” |
| Study size | 10 | Explain how the study size was arrived at | 8 | “We identified a cohort of patients 50-85 years of age who had at least one clinic-based visit at a KPSC medical facility between January 2008 and June 2018.”  “Patients were censored at the time of gastric cancer diagnosis, 85 years of age, end of 5-year follow-up period, end of study period, disenrollment from health plan, or death.” |

Continued on next page

| Quantitative variables | 11 | Explain how quantitative variables were handled in the analyses. If applicable, describe which groupings were chosen and why | 10 | “Weight change from 1-year prior (categorized as either gain of over 10 pounds, gain between 0-10 pounds, loss of 0.1-10 pounds, or loss of over 10 pounds) was reported but not included as potential predictor, because weight loss is considered a late symptom of cancer.” |
| --- | --- | --- | --- | --- |
| Statistical methods | 12 | (*a*) Describe all statistical methods, including those used to control for confounding | 10-11 | “The entire dataset was split into two: records from one KPSC medical service area were excluded from initial analysis and used exclusively for testing of the final model, while those from all other KPSC medical service areas (n=12) were included for training and validation. Predictive models for gastric cancer were developed using the cross-validated Lasso regression.(16) Through a regularization term, LASSO regression models can select the most important features and thus minimize overfitting. Out of the 50 models derived from the 50 training datasets (10 imputation datasets x 5-fold cross validation), the model that appeared the most often was selected as the final model.  For validation, the algorithm of the final model was applied to the remaining validation datasets that were previously excluded. There was no overlap between training and validation datasets. The algorithm was also applied on the 10 testing datasets. The discriminative power was evaluated by c-index, averaged across all the relevant validation datasets for cohort members. Calibration was assessed by calibration plots with five risk groups (<50th, 50–74th, 75–90th, 90–94th, 95–100th percentiles). A calibration plot was produced for each winning model.” |
|  |  | (*b*) Describe any methods used to examine subgroups and interactions | 11 | “To assess the potential clinical utility of the final model, we estimated the incidence of gastric cancer among patients based on traditionally defined risk factors (e.g., family history, history of H. pylori) compared to those based on model-predicted risk. Specifically, we examined the following: incidence among all patients 50-75 years of age, percent true cases in all gastric cancer patients, 5-year incidence rate and 95% CI, time to cancer (in cancer patients only) and number needed to screen to detect one single case.” |
|  |  | (*c*) Explain how missing data were addressed | 11 | “Missing data  ‘missRanger’ was applied to impute the missing values if the frequency of missing for a feature was less than 60%. We used predictive mean matching method with k=3. Laboratory measures or weight-related features with 60% or more missingness or change/change rate measures with 80% or more missingness were not included in the model development process. Ten imputed datasets were generated.” |
|  |  | (*d*) *Cohort study*—If applicable, explain how loss to follow-up was addressed  *Case-control study*—If applicable, explain how matching of cases and controls was addressed  *Cross-sectional study*—If applicable, describe analytical methods taking account of sampling strategy | 8 | “Patients were censored at the time of gastric cancer diagnosis, 85 years of age, end of 5-year follow-up period, end of study period, disenrollment from health plan, or death.” |
|  |  | (*e*) Describe any sensitivity analyses | 11 | “To assess the potential clinical utility of the final model, we estimated the incidence of gastric cancer among patients based on traditionally defined risk factors (e.g., family history, history of H. pylori) compared to those based on model-predicted risk. Specifically, we examined the following: incidence among all patients 50-75 years of age, percent true cases in all gastric cancer patients, 5-year incidence rate and 95% CI, time to cancer (in cancer patients only) and number needed to screen to detect one single case.” |
| Results | | | | |
| Participants | 13* | (a) Report numbers of individuals at each stage of study—eg numbers potentially eligible, examined for eligibility, confirmed eligible, included in the study, completing follow-up, and analysed | Fig. 1 | Fig. 1 |
|  |  | (b) Give reasons for non-participation at each stage | Fig. 1 | Fig. 1 |
|  |  | (c) Consider use of a flow diagram | Fig. 1 | Fig. 1 |
| Descriptive data | 14* | (a) Give characteristics of study participants (eg demographic, clinical, social) and information on exposures and potential confounders | 13 | Table 1 |
|  |  | (b) Indicate number of participants with missing data for each variable of interest | 13 | Table 1 |
|  |  | (c) *Cohort study*—Summarise follow-up time (eg, average and total amount) | 15 | Table 2 |
| Outcome data | 15* | *Cohort study*—Report numbers of outcome events or summary measures over time | 15 | Table 2 |
|  |  | *Case-control study—*Report numbers in each exposure category, or summary measures of exposure | N/A | N/A |
|  |  | *Cross-sectional study—*Report numbers of outcome events or summary measures | N/A | N/A |
| Main results | 16 | (*a*) Give unadjusted estimates and, if applicable, confounder-adjusted estimates and their precision (eg, 95% confidence interval). Make clear which confounders were adjusted for and why they were included | 15, 18 | Table 2 (page 15),  Table 3 (page 18) |
|  |  | (*b*) Report category boundaries when continuous variables were categorized | 13, 15 | Table 1 (page 13),  Table 2 (page 15) |
|  |  | (*c*) If relevant, consider translating estimates of relative risk into absolute risk for a meaningful time period | N/A | N/A |

Continued on next page

| Other analyses | 17 | Report other analyses done—eg analyses of subgroups and interactions, and sensitivity analyses | N/A | N/A |
| --- | --- | --- | --- | --- |
| Discussion | | | | |
| Key results | 18 | Summarise key results with reference to study objectives | 19 | “Based on data from a racially/ethnically diverse, regional integrated health care system in the United States, we developed a risk prediction model for gastric cancer. The model included a total of 9 routinely available parameters: age, sex, racial/ethnic background, family history, smoking status, H. pylori infection, PPI usage, as well as HGB value. These factors can be easily obtained from patient interview or abstracted from the electronic medical record. The prediction tool demonstrated a potentially more efficient approach to targeted screening for gastric cancer compared to reliance on traditional risk factors.” |
| Limitations | 19 | Discuss limitations of the study, taking into account sources of potential bias or imprecision. Discuss both direction and magnitude of any potential bias | 20 | “The present study has several limitations. Given the retrospective nature of the study, we were unable to evaluate the role of diet in determining risk of gastric cancer as certain types of diet including preserved foods or nitrates is an established risk factor for development of gastric cancer.(21) We did not include histologic data such as presence of atrophic gastritis or intestinal metaplasia as part of our model, as our model is intended to be applied as a pre-endoscopic tool in decision-making regarding initiation of screening.” |
| Interpretation | 20 | Give a cautious overall interpretation of results considering objectives, limitations, multiplicity of analyses, results from similar studies, and other relevant evidence | 21 | “In conclusion, we have developed a prediction model for gastric cancer based on data from the United States within a diverse racial/ethnic and socioeconomic population. Application of the model may be used to identify patients who are at higher risk for gastric cancer within a 5-year time frame representing a reasonable time frame to undergo endoscopic testing and surveillance. Such an approach to patient selection may be more widely applicable with the widespread adoption of electronic health systems providing an opportunity for targeted screening and prevention for gastric cancer in low prevalence regions such as the United States.” |
| Generalisability | 21 | Discuss the generalisability (external validity) of the study results | 19-21 | “Based on data from a racially/ethnically diverse, regional integrated health care system in the United States, we developed a risk prediction model for gastric cancer. The model included a total of 9 routinely available parameters: age, sex, racial/ethnic background, family history, smoking status, H. pylori infection, PPI usage, as well as HGB value. These factors can be easily obtained from patient interview or abstracted from the electronic medical record… Application of the model may be used to identify patients who are at higher risk for gastric cancer within a 5-year time frame representing a reasonable time frame to undergo endoscopic testing and surveillance. Such an approach to patient selection may be more widely applicable with the widespread adoption of electronic health systems providing an opportunity for targeted screening and prevention for gastric cancer in low prevalence regions such as the United States.” |
| Other information | |  | | |
| Funding | 22 | Give the source of funding and the role of the funders for the present study and, if applicable, for the original study on which the present article is based | 2 | “Financial support: The project described was supported, in part, by the Department of Research and Evaluation within the Southern California Permanente Medical Group with funds from the Medical Group and the Kaiser Permanente Community Benefit Fund.” |

*Give information separately for cases and controls in case-control studies and, if applicable, for exposed and unexposed groups in cohort and cross-sectional studies.

**Note:** An Explanation and Elaboration article discusses each checklist item and gives methodological background and published examples of transparent reporting. The STROBE checklist is best used in conjunction with this article (freely available on the Web sites of PLoS Medicine at http://www.plosmedicine.org/, Annals of Internal Medicine at http://www.annals.org/, and Epidemiology at http://www.epidem.com/). Information on the STROBE Initiative is available at www.strobe-statement.org.
