# Supplementary material for: Stomach Cancer Prediction Model (SCoPM): An approach to risk stratification in a diverse U.S. population
Source: PLoS One. 2024 May 21;19(5):e0303153. doi: 10.1371/journal.pone.0303153 (PMC11108155; doi:10.1371/journal.pone.0303153)
Supplement: S1 Fig — (DOCX) [file pone.0303153.s002.docx]

**Preparation of training**

DS6

DS5

DS2

DS1

DS4

DS3

DS7

DS8

DS9

DS1A

DS1B

DS1C

DS1D

DS1E

DS5A

DS5B

DS5C

DS5D

DS5E

DS6A

DS6B

DS6C

DS6D

DS6E

DS7A

DS7B1B

DS7C

DS&D

DS7E

DS8A

DS8B

DS8C

DS8D

DS8EE

DS9A

DS9B

DS9C

DS9D

DS9E

DS4A

DS4B

DS4C

DS4D

DS4E

DS3A

DS3B

DS3C

DS3D

DS1E

DS2A

DS2B

DS2C

DS2D

DS2E

Imputation process

Original DS

**and validation datasets**

DS10E

DS10D

DS10C

DS10B

DS10A

DS10
