# Supplementary material for: Stomach Cancer Prediction Model (SCoPM): An approach to risk stratification in a diverse U.S. population
Source: PLoS One. 2024 May 21;19(5):e0303153. doi: 10.1371/journal.pone.0303153 (PMC11108155; doi:10.1371/journal.pone.0303153)
Supplement: S1 File — (DOCX) [file pone.0303153.s003.docx]

# Supporting information

**Table S1. Variable included in the analyses.**

| **Variables** | **Definitions** | **Included as potential predictor** |
| --- | --- | --- |
| Age | Calculated as of the index date based on self-reported date of birth | Yes |
| Sex | Self-reported male/female | Yes |
| Race/ethnicity | Based on patients' self-reporting: Categorized as non-Hispanic White, non-Hispanic Black, Hispanic, non-Hispanic Asian/Pacific Islander, and other | Yes |
| History of H. pylori infection | Based on encounter diagnosis (ICD-9 041.86 and ICD-10 B96.81), problem list (ICD-9 041.86 and ICD-10 B96.81), medical history, and also lab tests (serum antibody test CPT 86677 and stool antigen test 87338). Value: Yes/no. | Yes |
| History of gastric ulcer | Based on encounter diagnosis codes, problem list, and medical history table with ICD-9 531.x or ICD-10 K25.x. KPSC internal diagnosis codes (121139731, 121139735) were also used. Value: Yes/no. | Yes |
| Family history of gastric cancer | Obtained through KPSC family history table and supplemented by KPSC problem list with internal diagnosis code 12147180. Value: Yes/no. | Yes |
| Smoking | Obtained through reporting from direct clinical ascertainment. Most recent record within 1-year prior to or as of the index date was retrieved. Categorized as never smoker, former smoker, current smoker, or unknown. | Yes |
| Drinking | Obtained through reporting from direct clinical ascertainment. Most recent record within 1-year prior to or as of index date was retrieved. Categorized as yes, no, or unknown. | Yes |
| BMI | Based on vitals during clinic-based visits. Most recent record within 6-month prior to or as of index date was retrieved. Categorized as underweight (less than 18.5 kg/m2), normal (18.5 to 25 kg/m2), overweight (25 to 30 kg/m2), obesity (30 or higher kg/m2), and unknown. | Yes |
| Weight change | Weight change from 1-year prior (weight at baseline - weight at 1-year prior). Baseline weight was the most recent measurement within 6 months prior to or as of the index date. The one measured closest to 12 months prior to index date within 9-15 mos. prior to the index date was obtained as the weight at 1 year prior. Categorized as either gain of over 4.5 kg (10 pounds), gain between 0 – 4.5 kg (including no change in weight), loss of 4.5 kg or less, or loss of over 4.5 kg. | No |
| Use of proton-pump inhibitor (PPI) therapy | Extracted from outpatient pharmacy records. Included dexlansoprazole, esomeprazole, lansoprazole, omeprazole, pantoprazole, and rabeprazole. Grouped into no use, new-onset use (within 6 months), or chronic use (beyond 6 months). |  |
| Baseline hemoglobin | Extracted from lab records. Most recent record within 6 months prior to or as of index date was retrieved. Categorized as normal (female 12-16; male 14-18), abnormal, and unknown. | Yes |
| Hemoglobin change | Change from 1-year prior (baseline hemoglobin - hemoglobin at 1 year prior). Baseline hemoglobin was defined as above. The one measured closest to 12-month prior to index date within 9-15 mos. prior to the index date was obtained as the hemoglobin at 1-year prior. Continuous measure. | Yes |
| Baseline Mean Corpuscular Volume (MCV) | Extracted from lab records. Most recent record within 6-month prior to or as of index date was retrieved. Categorized as normal (81-99), abnormal and unknown. | Yes |
| MCV change | Change from 1-year prior (baseline MCV - MCV at 1-year prior). Baseline MCV was defined as above. The one measured closest to 12-month prior to index date within 9-15 mos. prior to index date was obtained as the MCV at 1-year prior. Continuous measure. | Yes |
| Gastrointestinal (GI) symptoms* | Obtained through smart phrases with EHR and ICD-9 and ICD-10 codes (when applicable).  Classified as absence of symptoms (asymptomatic), present within 6 months, or present beyond 6 months. | No |
| BMI, body mass index; CPT, Current Procedural Terminology; ICD, International Classification of Diseases; KPSC, Kaiser Permanente Southern California; MCV, mean corpuscular volume.  *Includes abdominal bloating, abdominal pain, anorexia, dyspepsia, dysphagia, early satiety, fatigue, gastroesophageal reflux disease, melena, nausea, weight loss. | | |

**Table S2. More details on preparation of training and validation datasets. Refer to Figure S1 for descriptions of dataset names.**

| **Training and validation dataset** | **Training dataset** | **Validation dataset** |
| --- | --- | --- |
| 1 | All subsets in DS1 except for DS1A | DS1A |
| 2 | All subsets in DS1 except for DS1B | DS1B |
| 3 | All subsets in DS1 except for DS1C | DS1C |
| 4 | All subsets in DS1 except for DS1D | DS1D |
| 5 | All subsets in DS1 except for DS1E | DS1E |
| 6 | All subsets in DS2 except for DS2A | DS2A |
| 7 | All subsets in DS2 except for DS2B | DS2B |
| 8 | All subsets in DS2 except for DS2C | DS2C |
| 9 | All subsets in DS2 except for DS2D | DS2D |
| 10 | All subsets in DS2 except for DS2E | DS2E |
| 11 | All subsets in DS3 except for DS3A | DS3A |
| 12 | All subsets in DS3 except for DS3B | DS3B |
| 13 | All subsets in DS3 except for DS3C | DS3C |
| 14 | All subsets in DS3 except for DS3D | DS3D |
| 15 | All subsets in DS3 except for DS3E | DS3E |
| 16 | All subsets in DS4 except for DS4A | DS4A |
| 17 | All subsets in DS4 except for DS4B | DS4B |
| 18 | All subsets in DS4 except for DS4C | DS4C |
| 19 | All subsets in DS4 except for DS4D | DS4D |
| 20 | All subsets in DS4 except for DS4E | DS4E |
| 21 | All subsets in DS5 except for DS5A | DS5A |
| 22 | All subsets in DS5 except for DS5B | DS5B |
| 23 | All subsets in DS5 except for DS5C | DS5C |
| 24 | All subsets in DS5 except for DS5D | DS5D |
| 25 | All subsets in DS5 except for DS5E | DS5E |
| 26 | All subsets in DS6 except for DS6A | DS6A |
| 27 | All subsets in DS6 except for DS6B | DS6B |
| 28 | All subsets in DS6 except for DS6C | DS6C |
| 29 | All subsets in DS6 except for DS6D | DS6D |
| 30 | All subsets in DS6 except for DS6E | DS6E |
| 31 | All subsets in DS7 except for DS7A | DS7A |
| 32 | All subsets in DS7 except for DS7B | DS7B |
| 33 | All subsets in DS7 except for DS7C | DS7C |
| 34 | All subsets in DS7 except for DS7D | DS7D |
| 35 | All subsets in DS7 except for DS7E | DS7E |
| 36 | All subsets in DS8 except for DS8A | DS8A |
| 37 | All subsets in DS8 except for DS8B | DS8B |
| 38 | All subsets in DS8 except for DS8C | DS8C |
| 39 | All subsets in DS8 except for DS8D | DS8D |
| 40 | All subsets in DS8 except for DS8E | DS8E |
| 41 | All subsets in DS9 except for DS9A | DS9A |
| 42 | All subsets in DS9 except for DS9B | DS9B |
| 43 | All subsets in DS9 except for DS9C | DS9C |
| 44 | All subsets in DS9 except for DS9D | DS9D |
| 45 | All subsets in DS10 except for DS9E | DS10E |
| 46 | All subsets in DS10 except for DS9A | DS10A |
| 47 | All subsets in DS10 except for DS9B | DS10B |
| 48 | All subsets in DS10 except for DS9C | DS10C |
| 49 | All subsets in DS10 except for DS9D | DS10D |
| 50 | All subsets in DS10 except for DS9E | DS10E |
| **Testing dataset (held-out dataset)** | **Testing dataset name** | |
| 1 | DS11 | |
| 2 | DS12 | |
| 3 | DS13 | |
| 4 | DS14 | |
| 5 | DS15 | |
| 6 | DS16 | |
| 7 | DS17 | |
| 8 | DS18 | |
| 9 | DS19 | |
| 10 | DS20 | |

**Table S3. Symptoms, presented as N (%), N=1,844,643.**

| **Symptom** | **Value** |
| --- | --- |
| **Abdominal bloating** |  |
| Within 6 mos. | 15,769 (0.9%) |
| Beyond 6 mos. | 87,224 (4.7%) |
| No symptom | 1,741,650 (94.4%) |
| **Abdominal pain** |  |
| Within 6 mos. | 177,589 (9.6%) |
| Beyond 6 mos. | 565,765 (30.7%) |
| No symptom | 1,101,289 (59.7%) |
| **Anorexia** |  |
| Within 6 mos. | 4,958 (0.3%) |
| Beyond 6 mos. | 15,955 (0.9%) |
| No symptom | 1,823,730 (98.9%) |
| **Dyspepsia** |  |
| Within 6 mos. | 26,679 (1.4%) |
| Beyond 6 mos. | 183,991 (10%) |
| No symptom | 1,633,973 (88.6%) |
| **Dysphagia** |  |
| Within 6 mos. | 18,339 (1%) |
| Beyond 6 mos. | 75,945 (4.1%) |
| No symptom | 1,750,359 (94.9%) |
| **Early satiety** |  |
| Within 6 mos. | 1,056 (0.1%) |
| Beyond 6 mos. | 3,586 (0.2%) |
| No symptom | 1,840,001 (99.7%) |
| **Fatigue** |  |
| Within 6 mos. | 180,665 (9.8%) |
| Beyond 6 mos. | 498,907 (27%) |
| No symptom | 1,165,071 (63.2%) |
| **Gastroesophageal reflux disease** |  |
| Within 6 mos. | 196,018 (10.6%) |
| Beyond 6 mos. | 359,906 (19.5%) |
| No symptom | 1,288,719 (69.9%) |
| **Melena** |  |
| Within 6 mos. | 16,617 (0.9%) |
| Beyond 6 mos. | 68,653 (3.7%) |
| No symptom | 1,759,373 (95.4%) |
| **Nausea** |  |
| Within 6 mos. | 113,403 (6.1%) |
| Beyond 6 mos. | 379,866 (20.6%) |
| No symptom | 1,351,374 (73.3%) |
| **Weight loss** |  |
| Within 6 mos. | 38,459 (2.1%) |
| Beyond 6 mos. | 119,472 (6.5%) |
| No symptom | 1,686,712 (91.4%) |

**Table S4. Baseline patient characteristics. N (%) unless otherwise specified. N=1,844,643.**

| **Patient Characteristics** | **Training/validation dataset (N=1,555,392)** | **Testing dataset (N=289,251)** |
| --- | --- | --- |
| **Age in years, mean (SD)** | 61.9 (9.35) | 62.2 (9.45) |
| 50-59 | 779,687 (50.1%) | 140,877 (48.7%) |
| 60-69 | 456,349 (29.3%) | 86,795 (30%) |
| 70+ | 319,356 (20.5%) | 61,579 (21.3%) |
| **Sex** |  |  |
| Female | 828,279 (53.3%) | 154,024 (53.2%) |
| Male | 727,113 (46.7%) | 135,227 (46.8%) |
| **Race/ethnicity** |  |  |
| Asian/Pacific Islander | 172,795 (11.1%) | 26,140 (9%) |
| Black | 160,379 (10.3%) | 13,828 (4.8%) |
| Hispanic | 493,875 (31.8%) | 60,345 (20.9%) |
| White | 655,107 (42.1%) | 175,499 (60.7%) |
| Other/unknown | 73,236 (4.7%) | 13,439 (4.7%) |
| **History of H. pylori infection** | 100,288 (6.4%) | 12,747 (4.4%) |
| **History of gastric ulcer** | 28,845 (1.9%) | 4,664 (1.6%) |
| **Family history of gastric cancer** | 33,805 (2.2%) | 5,276 (1.8%) |
| **Smoking** |  |  |
| Non-smoker | 914,335 (58.8%) | 158,875 (54.9%) |
| Former smoker | 419,693 (27%) | 83,135 (28.7%) |
| Current smoker | 130,406 (8.4%) | 23,781 (8.2%) |
| Unknown | 90,958 (5.8%) | 23,460 (8.1%) |
| **Drinking** |  |  |
| No | 739,081 (47.5%) | 103,556 (35.8%) |
| Yes | 562,929 (36.2%) | 106,687 (36.9%) |
| Unknown | 253,382 (16.3%) | 79,008 (27.3%) |
| **BMI, mean (SD)** | 29.2 (6.17) | 28.7 (6.03) |
| Underweight | 15,258 (1%) | 3,043 (1.1%) |
| Normal | 344,093 (22.1%) | 69,307 (24%) |
| Overweight | 528,234 (34%) | 95,762 (33.1%) |
| Obesity | 544,044 (35%) | 88,454 (30.6%) |
| Unknown | 123,763 (8%) | 32,685 (11.3%) |
| **Weight change*** | N=1,019,390 | N=173,364 |
| Median (Q1, Q3) | 0 (-4.8, 4.2) | 0 (-5.0, 4.2) |
| Weight gain > 4.5 kg (10 lbs.) | 90,694 (8.9%) | 16,177 (9.3%) |
| Weight gain ≤ 4.5 kg or no change | 429,937 (42.2%) | 72,836 (42%) |
| Weight loss ≤ 4.5 kg | 389,409 (38.2%) | 64,884 (37.4%) |
| Weight loss > 4.5 kg | 109,350 (10.7%) | 19,467 (11.2%) |
| **Proton pump inhibitors** |  |  |
| No PPI | 1,124,258 (72.3%) | 217,732 (75.3%) |
| PPI initiated within 6 mos. | 396,164 (25.5%) | 66,143 (22.9%) |
| PPI initiated beyond 6 mos. | 34,970 (2.2%) | 5,376 (1.9%) |
| **Lab** |  |  |
| **Baseline hemoglobin** | N=748,068 | N=137,901 |
| Median (Q1, Q3) | 13.7 (12.7, 14.7) | 13.8 (12.8, 14.7) |
| Normal | 549,409 (35.3%) | 103,506 (35.8%) |
| Abnormal | 198,659 (12.8%) | 34,395 (11.9%) |
| Not tested | 807,324 (51.9%) | 151,350 (52.3%) |
| **Hemoglobin change** | N=337,842 | N=59,634 |
| Median (Q1, Q3) | -0.1 (-0.7, 0.5) | 0 (-0.6, 0.5) |
| **Baseline MCV** | N=732,819 | N=135,686 |
| Median (Q1, Q3) | 90.4 (87.2, 93.5) | 91.5 (88.4, 94.6) |
| Normal | 663,847 (42.7%) | 121,929 (42.2%) |
| Abnormal | 68,972 (4.4%) | 13,757 (4.8%) |
| Not tested | 822,573 (52.9%) | 153,565 (53.1%) |
| **MCV change** | N=325,019 | N=57,831 |
| Median (Q1, Q3) | 0 (-1.4, 1.5) | 0 (-1.4, 1.4) |

BMI, body mass index; MCV, mean corpuscular volume.

*Weight change is defined as the change in weight within 1 year prior to baseline (baseline – 1 year prior).

**Table S5. Baseline characteristics of gastric cancer patients. N (%) unless otherwise specified. N=994.**

| **Patient Characteristics** | **Value** |
| --- | --- |
| **Age at gastric cancer diagnosis, years** |  |
| Mean (SD) | 71.1 (9.1) |
| Median (Q1, Q3) | 72.3 (64.1, 78.7) |
| **Sex** |  |
| Female | 391 (39.3%) |
| Male | 603 (60.7%) |
| **Race/ethnicity** |  |
| Asian/Pacific Islander | 121 (12.2%) |
| Black | 133 (13.4%) |
| Hispanic | 344 (34.6%) |
| White | 383 (38.5%) |
| Other/Unknown | 13 (1.3%) |
| **AJCC stage** |  |
| 0 | 9 (0.9%) |
| 1 | 165 (16.6%) |
| 2 | 93 (9.4%) |
| 3 | 93 (9.4%) |
| 4 | 331 (33.3%) |
| Unknown | 303 (30.5%) |
| **SEER stage** |  |
| In situ | 5 (0.5%) |
| Localized | 277 (27.9%) |
| Regional | 204 (20.5%) |
| Distant | 382 (38.4%) |
| Unknown | 126 (12.7%) |
| **Site** |  |
| Body of stomach | 147 (14.8%) |
| Cardia NOS, GE junction | 257 (25.9%) |
| Fundus of stomach | 46 (4.6%) |
| Gastric antrum | 128 (12.9%) |
| Greater curvature of stomach, NOS | 33 (3.3%) |
| Lesser curvature of stomach, NOS | 74 (7.4%) |
| Overlapping lesion of stomach | 56 (5.6%) |
| Pylorus | 25 (2.5%) |
| Stomach, NOS | 153 (15.4%) |
| Unknown | 75 (7.5%) |
| **Histology** |  |
| Adenocarcinoma in adenomatous polyp | 10 (1%) |
| Adenocarcinoma in situ | 1 (0.1%) |
| Adenocarcinoma in situ in adenomatous polyp | 2 (0.2%) |
| Adenocarcinoma in situ in tubulovillous adenoma | 1 (0.1%) |
| Adenocarcinoma in tubulovillous adenoma | 5 (0.5%) |
| Adenocarcinoma in villous adenoma | 1 (0.1%) |
| Adenocarcinoma with mixed subtypes | 24 (2.4%) |
| Adenocarcinoma with neuroendocrine differentiation | 1 (0.1%) |
| Adenocarcinoma, intestinal type | 41 (4.1%) |
| Adenocarcinoma, NOS | 435 (43.8%) |
| Adenosquamous carcinoma | 5 (0.5%) |
| Atypical carcinoid tumor | 4 (0.4%) |
| Carcinoid tumor, malignant | 50 (5%) |
| Carcinoma in situ, NOS | 1 (0.1%) |
| Carcinoma, diffuse type | 11 (1.1%) |
| Carcinoma, NOS | 21 (2.1%) |
| Gastrointestinal stromal sarcoma | 60 (6%) |
| Large cell neuroendocrine carcinoma | 1 (0.1%) |
| Leiomyosarcoma, NOS | 1 (0.1%) |
| Linitis plastica | 3 (0.3%) |
| Mucin-producing adenocarcinoma | 2 (0.2%) |
| Mucinous adenocarcinoma | 16 (1.6%) |
| Neoplasm, malignant | 3 (0.3%) |
| Neuroendocrine carcinoma | 9 (0.9%) |
| Plasmablastic lymphoma | 1 (0.1%) |
| Signet ring cell carcinoma | 203 (20.4%) |
| Squamous cell carcinoma, NOS | 3 (0.3%) |
| Tubular adenocarcinoma | 4 (0.4%) |
| Unknown | 75 (7.5%) |

AJCC, American Joint Committee on Cancer; GE, gastroesophageal; NOS, not otherwise specified; SEER, Surveillance, Epidemiology, and End Results Program.
